# THOR methylation as a pan-cancer mechanism of *TERT* activation: toward a clinically relevant epigenetic biomarker

**DOI:** 10.3389/fonc.2026.1893675

**Published:** 2026-07-17

**Authors:** Alain Chebly, Edith Chevret

**Affiliations:** 1 Center Jacques Loiselet for Medical Genetics and Genomics (CGGM), Faculty of Medicine, Saint Joseph University of Beirut (USJ), Beirut, Lebanon; 2 Bordeaux Institute of Oncology (BRIC), INSERM 1312, Université de Bordeaux, Bordeaux, France

**Keywords:** cancer biomarker, epigenetic activation, hypermethylation, oncogenesis, telomerase, TERT, THOR

## Abstract

Telomerase reverse transcriptase (*TERT*) activation is a hallmark of cancer and results from multiple genetic and epigenetic mechanisms. Among these, methylation of the TERT Hypermethylated Oncological Region (THOR) has emerged as a paradoxical epigenetic mechanism associated with increased *TERT* expression across multiple cancers. This mini-review summarizes the current evidence on the biological role, molecular mechanisms, and clinical significance of THOR methylation in both solid and hematological cancers. We discuss how THOR differs from broader *TERT* promoter methylation, its interaction with other mechanisms of *TERT* activation, and its potential role as a diagnostic, prognostic, and minimally invasive biomarker. We also highlight current limitations, including methodological heterogeneity and the need for assay standardization and prospective clinical validation. Although therapeutic targeting of THOR remains investigational, advances in locus-specific epigenome editing may offer future opportunities for selective modulation of TERT expression. Overall, accumulating evidence supports THOR methylation as a promising biomarker and an important contributor to the complex regulation of telomerase activation in cancer.

## Introduction

Telomerase activation, primarily driven by re-expression of its catalytic subunit *TERT*, is recognized as a key enabling step in oncogenesis (1). Recurrent *TERT* promoter (*TERTp*) mutations (C228T and C250T) have been identified in several malignancies and represent a well-established mechanism of telomerase activation (2). However, many cancers reportedly exhibit telomerase activity in the absence of these mutations, suggesting alternative regulatory mechanisms (3). This discrepancy between telomerase activity and the absence of *TERT* promoter mutations represents a critical gap in our understanding of telomerase regulation in cancer, highlighting the need to identify alternative mechanisms such as epigenetic regulation. Among these, epigenetic regulation of the *TERT* locus has gained increasing attention, particularly the *TERT* Hypermethylated Oncological Region (THOR) (4).

We propose that THOR methylation represents a central and underrecognized mechanism of telomerase activation across cancers, particularly in *TERTp* mutation–negative tumors.

## THOR: a paradoxical epigenetic mechanism

**Figure 1:**
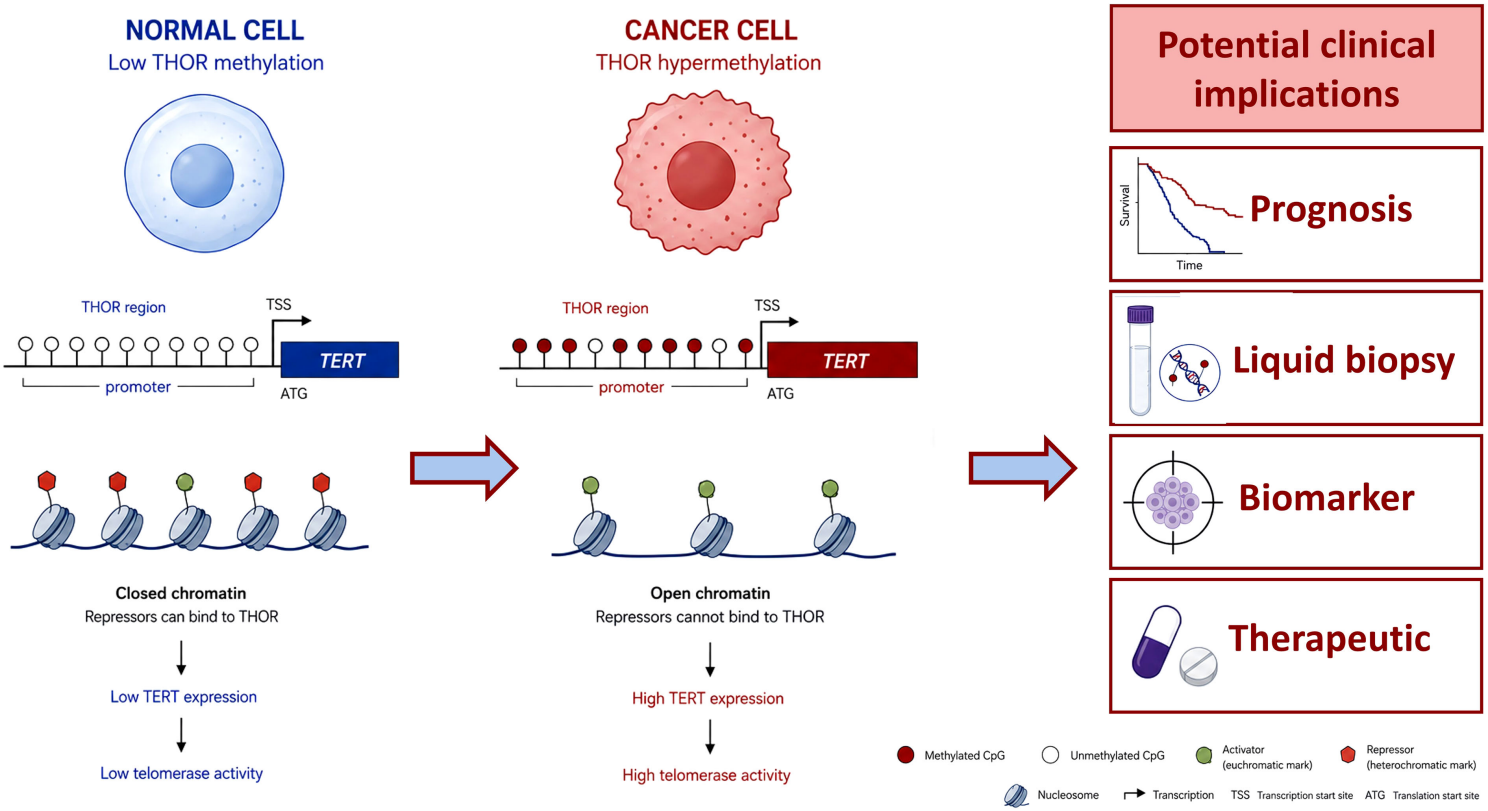
Proposed model of THOR-mediated epigenetic regulation of TERT expression and its potential clinical implications.

THOR is a CpG-rich region located upstream of the TERT core promoter. A dual-methylation pattern has been described comprising both hypermethylated and hypomethylated regions. Unlike classical promoter methylation, which is associated with gene silencing, hypermethylation of THOR has been consistently associated with increased *TERT* expression (4, 5). One proposed mechanism suggests that THOR hypermethylation prevents the binding of transcriptional repressors, thereby maintaining an open chromatin configuration permissive for *TERT* transcription (5, 6) (**Figure 1**).

Earlier studies investigating *TERT* promoter methylation often assessed the promoter as a single regulatory unit, resulting in apparently conflicting observations regarding the relationship between promoter methylation and gene expression. The identification of THOR demonstrated that methylation of the distal upstream promoter region has distinct biological consequences from methylation immediately surrounding the transcription start site (TSS). Whereas methylation within THOR is generally associated with increased *TERT* transcription through the inhibition of transcriptional repressors, the proximal promoter surrounding the TSS typically remains hypomethylated, allowing recruitment of the transcriptional machinery and maintenance of an open chromatin configuration (5).

Beyond these regional differences, the functional consequences of THOR also depend on CpG-specific methylation patterns. Hypermethylation within THOR has been proposed to inhibit the binding of transcriptional repressors, including CTCF, thereby relieving transcriptional repression of *TERT*. At the same time, maintenance of an accessible chromatin configuration at the proximal promoter, together with active histone modifications, facilitates RNA polymerase II recruitment and transcriptional activation. This dual epigenetic configuration provides a mechanistic explanation for the paradoxical association between distal promoter hypermethylation and increased *TERT* expression observed across multiple malignancies (7).

## Evidence across cancers: a pan-cancer signal

Accumulating evidence, across multiple studies, demonstrates that THOR methylation is present across a wide range of malignancies, including gliomas, hepatocellular carcinoma, prostate cancer, thyroid cancer, gastric cancer, and hematological malignancies (4, 7–13). As summarized in **Table 1**, the evolution of evidence from early paradoxical methylation observations to THOR-defined studies supports a progressive consolidation of this mechanism across cancers. Importantly:

**Table 1:** Chronological synthesis of studies evaluating THOR methylation across human malignancies, including study characteristics, methodological approaches, principal molecular findings, and reported clinical associations.

Era	Study	Disease/model	Cohort/sample type	Assay/CpG coverage	TERTp mutation status	TERT expression/telomerase activity	Clinical/translational relevance
Pre-THOR	Devereux et al., 1999 (23).	Multiple cancer cell lines	37 cell lines	Methylation analysis of up to 72 CpGs from ~500 bp upstream of TSS into exon 1	NR	Methylation assessed in relation to TERT regulation	Early evidence that TERT promoter methylation may be involved in telomerase regulation
	Dessain et al., 2000 (24).	Multiple tumors/cell models	Primary tissues, non-immortalized cells, cultured cells and tumors	Methylation-specific PCR	NR	TERT CpG island methylation did not uniformly correlate with TERT expression	Demonstrated complexity of TERT promoter methylation regulation
	Nomoto et al., 2002 (25).	Colorectal cancer	13 cancer cell lines, 8 WBC samples, 24 CRC tissues	Bisulfite PCR-SSCP	NR	hTERT expression assessed by RT-PCR; hypermethylation associated with hTERT expression	Supported association between TERT promoter hypermethylation and expression in CRC
	Guilleret et al., 2002 (26).	Multiple cancers	Cell lines, tumor and normal tissues	Methylation analysis of TERT promoter	NR	Positive correlation between TERT promoter hypermethylation, TERT mRNA and telomerase activity	Key early evidence of paradoxical methylation–activation relationship
	Zinn et al., 2007 (6).	Multiple cancers	Cancer and immortalized cell lines	DNA methylation and chromatin profiling	NR	TERT expressed despite promoter methylation, with preserved unmethylated/active chromatin near TSS	Helped refine dual methylation model: distal methylation with proximal permissive chromatin
	Azouz et al., 2010 (27).	Acute promyelocytic leukemia	APL cell models	TERT promoter methylation analysis	NR	Retinoid response linked to epigenetic regulation of TERT	Suggested distal methylated promoter regions may regulate transcription factor binding and TERT repression
	Pettigrew et al., 2012 (28).	AML	AML cell lines	TERT methylation analysis; demethylating agent exposure	NR	TERT expression, telomerase activity, telomere length and cell death assessed	Explored relationship between TERT methylation and response to 5-aza-2′-deoxycytidine
Early THOR concept	Castelo-Branco et al., 2013 (4).	Childhood brain tumors	Pediatric brain tumor tissues	TERT promoter methylation analysis	NR	Integrated with molecular tumor features	Identified TERT promoter methylation as a risk-stratification biomarker
	Zhang X et al., 2015 (29).	AML	Cell lines and primary cells	TERT methylation analysis	NR	TERT methylation linked to telomerase activity; methylation remained unchanged after 5-aza treatment	Supported methylation-associated TERT activation in leukemia
	Zhang H et al., 2015 (10).	Hepatocellular carcinoma	HCC tumor and normal tissues	TERT promoter methylation analysis	NR	TERT methylation associated with TERT expression	Suggested association with HCC progression
	Castelo-Branco et al., 2016 (11).	Prostate cancer	Retrospective prostate cancer cohort	THOR/TERT promoter methylation signature	NR	Associated with TERT regulation	Predicted biochemical relapse; supported diagnostic/prognostic utility
	Wu et al., 2016 (8).	Gastric cancer	Gastric tumor and normal tissues	TERT methylation analysis	NR	Association with TERT expression reported	Associated with lymph node metastasis and prognosis
THOR defined	Lee et al., 2019 (5).	Pan-cancer	Cell lines, normal tissues, tumor tissues; pan-cancer tumor set	THOR methylation analysis	Evaluated with TERT promoter mutations	THOR hypermethylation associated with increased TERT expression	Defined THOR and proposed it as a prevalent telomerase-activating mechanism, independently or with TERTp mutations
THOR expansion	Garsuault et al., 2020 (7).	APL	APL cells/bone marrow/CD34+ models	DNA methylation with chromatin accessibility and histone mark analysis	NR	TERT expression assessed in relation to methylation and chromatin state	Showed complex relationship between methylation, chromatin accessibility, histone marks and hTERT expression
	Chebly et al., 2021 (9).	CTCL	CTCL cell lines, patient samples and healthy controls	Gene-specific TERT promoter methylation analysis; distal and proximal regions; demethylating agents	TERTp mutations uncommon	Distal hypermethylation from −650 to −150 bp and proximal hypomethylation from −150 to +150 bp associated with tumor cells	Demonstrated THOR-like methylation pattern in lymphoid malignancies. THOR distinguished malignant from benign cells.
	Lee et al., 2021 (30).	Multiple cancers	Cancer cell lines	Allele-specific DNA methylation analysis	Evaluated with genetic TERT alterations	Demonstrated allele-specific methylation effects on TERT expression	Refined model of THOR methylation and TERT activation
	Apolónio et al., 2022 (31).	Breast cancer	Discovery and validation breast cancer cohorts	Pyrosequencing of THOR	NR	THOR hypermethylation associated with higher TERT expression	THOR distinguished malignant from benign tissue and was proposed as biomarker/therapeutic target
	Kouroukli et al., 2023 (15).	Mature B-cell lymphomas	B-cell lymphoma cell lines/lymphoma entities	DNA methylation profiling of THOR and core promoter CpGs	Structural TERT alterations discussed	THOR methylation differed between lymphoma subtypes	Showed subtype-specific TERT promoter/THOR methylation patterns in mature B-cell lymphomas
	Li et al., 2024 (12).	Papillary thyroid cancer	PTC tissues/datasets	TERT promoter methylation analysis	Evaluated relative to TERT promoter mutation	TERT methylation associated with high TERT expression	Associated with poor clinical outcomes; potential prognostic marker
	Li et al., 2024 (32).	TERTp mutation–negative thyroid cancer	Thyroid cancer cell lines and tissues	TERT upstream promoter methylation analysis; demethylating agents and locus-specific demethylation	Focused on TERTp mutation–negative setting	Demethylation reduced TERT expression; functional evidence for methylation-mediated activation	Supported THOR/upstream methylation as an alternative activation mechanism and potential therapeutic vulnerability
	Seo et al., 2024 (33).	Papillary thyroid cancer	PTC tumor samples/bioinformatic analyses	TERT regulatory mechanism analysis including THOR	Compared TERTp-mutant and TERT-expressing TERTp-wild-type tumors	TERT expression independent of TERTp mutation associated with immune-enriched milieu	Supported alternative TERT activation mechanisms in PTC
	El Azzouzi et al., 2024 (34).	Bladder cancer	Bladder cancer patient tissues	THOR methylation and TERT promoter mutation analysis	60% TERTp mutations reported	88% THOR hypermethylation reported	Identified combined genetic and epigenetic TERT alterations as clinically relevant in bladder cancer

ALT, alternative lengthening of telomeres; APL, acute promyelocytic leukemia; CTCL, cutaneous T-cell lymphoma; HCC, hepatocellular carcinoma; NR, not reported; PTC, papillary thyroid carcinoma; TERT, telomerase reverse transcriptase; TERTp, TERT promoter; THOR, TERT Hypermethylated Oncological Region.

- THOR methylation is observed across diverse tumor types,
- It frequently occurs in tumors lacking *TERT* promoter mutations (TERTp) (9, 10, 12),
- And, it correlates with *TERT* expression and, in some contexts, with aggressive clinical behavior (5, 9, 11).

Collectively, these observations suggest that THOR methylation may represent a unifying epigenetic mechanism of *TERT* activation across cancers, particularly in tumors lacking canonical promoter mutations.

## Specific relevance in hematological malignancies

TERTp mutations are uncommon in most hematological malignancies (3, 14). In this context, THOR methylation may represent a dominant mechanism of telomerase activation. Studies in acute leukemias, lymphomas, and cutaneous T-cell lymphoma (CTCL) have demonstrated THOR methylation associated with *TERT* expression and disease progression (**Table 1**) (7, 9, 15). This observation is particularly relevant in hematological malignancies, where TERTp mutations are rare, suggesting that THOR methylation may represent a predominant mechanism of telomerase activation in these diseases.

## THOR within the landscape of *TERT* activation mechanisms

Although THOR methylation has emerged as an important epigenetic mechanism of *TERT* activation, it represents only one component of a broader landscape of telomerase regulation in cancer. Recurrent TERTp mutations create *de novo* binding sites for ETS transcription factors, resulting in constitutive *TERT* transcription and representing one of the most common genetic mechanisms of telomerase activation in several tumor types. In contrast, THOR hypermethylation provides an alternative epigenetic mechanism that may coexist with promoter mutations or contribute to *TERT* activation in promoter wild-type tumors. Additional mechanisms include copy-number gains and structural rearrangements involving the *TERT* locus, which can further enhance transcription by increasing gene dosage or repositioning strong regulatory enhancers near the promoter. Conversely, a subset of cancers maintains telomere length through the alternative lengthening of telomeres (ALT) pathway, a telomerase-independent mechanism frequently associated with alterations in *ATRX* or *DAXX*. Together, these observations indicate that THOR methylation should be viewed as part of a complex and complementary network of genetic and epigenetic mechanisms governing telomere maintenance rather than as a universal mechanism operating across all malignancies (2, 3, 16).

## Clinical implications

THOR methylation may help explain telomerase activation in tumors classified as “TERTp wild type,” thereby refining molecular stratification. THOR methylation represents a promising biomarker candidate:

- Diagnostic biomarker: distinguishing malignant from normal tissues (4, 9).
- Prognostic marker: association with tumor aggressiveness (8, 11).
- Potential liquid biopsy target: detection in circulating tumor DNA (ctDNA) represents an emerging concept (17, 18). Given its epigenetic nature, THOR methylation may be particularly suitable for detection in ctDNA, offering a promising avenue for non-invasive cancer diagnostics.

Although the available data support the feasibility of detecting THOR methylation in circulating cell-free DNA, its clinical implementation should be considered within the broader landscape of liquid biopsy technologies. Recent studies have demonstrated the growing value of plasma DNA methylation signatures and circulating tumor cell (CTC)-based monitoring for cancer detection, prognostication, and treatment response assessment (19, 20). Compared with these emerging approaches, THOR methylation offers the advantage of targeting a biologically relevant regulator of telomerase activation but still requires further analytical validation, assay standardization, and prospective clinical evaluation before routine clinical implementation. Future studies directly comparing THOR methylation with established liquid biopsy biomarkers may better define its complementary role in precision oncology.

The relationship between THOR methylation and *TERT* promoter mutations remains incompletely understood. Whether these mechanisms are mutually exclusive, cooperative, or context-dependent requires further investigation.

Several methodologies have been used to assess THOR methylation, including bisulfite sequencing, pyrosequencing, methylation-specific PCR, and targeted next-generation sequencing–based methylation approaches. However, variability in assay design, CpG coverage, and methylation thresholds across studies currently limits inter-study comparability and clinical implementation. Standardized and clinically validated assays will therefore be essential before THOR methylation can be integrated into routine molecular oncology workflows.

Although accumulating evidence supports THOR methylation as a promising diagnostic and prognostic biomarker across multiple malignancies, its therapeutic targeting remains largely investigational. Because THOR represents an epigenetic alteration rather than a genetic lesion, current approaches would rely on epigenetic modulators, including DNA methyltransferase inhibitors or locus-specific epigenome editing strategies. However, conventional epigenetic therapies induce widespread genome-wide methylation changes and may produce unintended effects on non-target genes, limiting their specificity. Future therapeutic strategies should therefore focus on more selective epigenetic editing approaches capable of modulating THOR methylation while minimizing off-target effects.

Beyond direct epigenetic modulation, emerging evidence suggests that *TERT* regulation is closely linked to broader cellular processes, including oxidative stress, cellular senescence, and NRF2-dependent signaling pathways. These interconnected mechanisms influence telomere maintenance, tumor persistence, and therapeutic response, highlighting the complexity of *TERT* regulation in cancer (21, 22). Although the precise relationship between THOR methylation and these pathways remains to be fully elucidated, integrating epigenetic biomarkers with cellular stress and senescence signaling may provide new opportunities for patient stratification and the development of more effective precision oncology strategies.

## Conclusion and future perspectives

THOR methylation represents an emerging and increasingly recognized mechanism of *TERT* activation across cancers. Its consistent association with telomerase expression, particularly in *TERT* promoter mutation–negative tumors, positions it as a potential unifying epigenetic biomarker.

Beyond its biological relevance, THOR methylation may help refine molecular classification of cancers and provide new opportunities for non-invasive diagnostics through liquid biopsy approaches. However, its clinical implementation will require further analytical validation, assay standardization, and prospective clinical studies.

Future research should focus on clarifying the interplay between THOR methylation and other mechanisms of *TERT* activation, including *TERT*p mutations, copy-number alterations, structural variants, and alternative lengthening of telomeres (ALT), while evaluating its prognostic and predictive value in large, well-characterized clinical cohorts. Integrating THOR methylation with genomic, transcriptomic, and other emerging biomarkers may further define its role in precision oncology.
